# Antimicrobial Activity of Cell-Free Supernatants Produced by Strains of Lactic Acid Bacteria Against *Staphylococcus aureus*

**DOI:** 10.3390/vetsci13020139

**Published:** 2026-01-30

**Authors:** Xinru Li, Yuejie Yang, Zijian Geng, Rui Wu, Shuai Lian, Jianfa Wang

**Affiliations:** 1 College of Animal Science and Veterinary Medicine, Heilongjiang Bayi Agricultural University, Daqing 163319, China; lixinru200101@163.com (X.L.); yangyuejie1997@163.com (Y.Y.); gengzijian_vet@126.com (Z.G.); 2 China Key Laboratory of Bovine Disease Control in Northeast China, Ministry of Agriculture and Rural Affairs, Daqing 163319, China; 3 Heilongjiang Provincial Key Laboratory of Prevention and Control of Bovine Diseases, Daqing 163319, China; 4 College of Biology and Agriculture, Jiamusi University, Jiamusi 154007, China; fuhewu@126.com

**Keywords:** lactic acid bacteria, *Staphylococcus aureus*, cell-free supernatant, antimicrobial activity

## Abstract

Bacterial zoonoses pose a serious threat to the development of animal husbandry, food safety, and public health. *Staphylococcus aureus* (*S. aureus*) is a major infectious and food-borne pathogen worldwide, and there was an urgent need to develop relevant methodologies for the control of bacterial infections. This study aimed to evaluate the effectiveness of cell-free supernatants (CFSs) produced by selected strains of *Lactiplantibacillus plantarum* (*L. plantarum*), *Lacticaseibacillus rhamnosus* (*L. rhamnosus*), *Streptococcus thermophilus* (*S. thermophilus*), and *Bifidobacterium longum* subspecies *infantis* (*B. infantis*) to inhibit in vitrogrown *S. aureus* BNCC 186335. CFSs of *S. thermophilus*, *B. infantis*, *L. plantarum,* and *L. rhamnosus* not only showed good antibacterial activity against *S. aureus* but also have strong stability and tolerance, which could destroy the integrity of cell membrane, lead to changes in cell morphology, and then strongly and rapidly kill bacteria. Notably, the primary antimicrobial substances in the CFSs of *L. plantarum* and *L. rhamnosus* were organic acids and protein components, whereas the main antimicrobial substances in the CFSs of *S. thermophilus* and *B. infantis* were organic acids. Meanwhile, four CFSs achieved substantial removal of biofilms and inhibited decreased ATP content. These findings suggest that the CFSs of *S. thermophilus*, *B. infantis*, *L. plantarum,* and *L. rhamnosus* may have potential applications as biological preservatives to control the contamination of *S. aureus* in the food industry and animal husbandry.

## Simple Summary

*S. aureus* infection is a significant player in causing negative impacts such as cow mastitis, and it is necessary to seek alternatives to antibiotics for intervention. Cell-free supernatants (CFSs) produced by probiotics have emerged as novel antimicrobial candidates attracting significant interest due to their dual functionality in safety and multiple beneficial health effects. This study demonstrates that the CFSs produced from the culture of *S. thermophilus*, *B. infantis*, *L. plantarum,* and *L. rhamnosus* strains have antibacterial activity against *S. aureus* BNCC 186335 cultured in vitro. Therefore, there is a possibility that these four CFSs could be used as natural antibacterial agents to prevent the occurrence of mastitis in dairy cows. This work further improves our understanding of how probiotics and their metabolites resist pathogenic bacteria and expands the scope of application of CFSs.

## 1. Introduction

Bacterial zoonoses are the most common and frequently occurring type among zoonoses, exerting a significant impact on food safety, human and animal health, as well as the development of animal husbandry [1]. Among them, *Staphylococcus aureus* is particularly important due to its global prevalence. First, *Staphylococcus aureus* (*S. aureus*) is an opportunistic pathogen that is widely colonized on the skin and in the digestive tract, mammary glands, and upper respiratory tract of animals [2]. *S. aureus* causes exudative dermatitis, bacterial chondronecrosis, mastitis, respiratory tract infections, and systemic diseases in livestock such as pigs, poultry, cattle, and sheep by causing pathogenic changes to the colonization site [3,4,5]. These diseases pose a great threat to animal health, welfare and productivity, and also pose a major challenge to food safety and public health. Second, *S. aureus*, as a widely present food-borne pathogen, is commonly attached to meat, fruit juices, milk, dairy products, and seafood [6]. *S. aureus*, present in fresh foods such as pork, chicken, fruit and seafood, exhibits strong adhesion to food and low sensitivity to antimicrobial agents through biofilm formation [7,8,9,10,11]. Meanwhile, *S. aureus* is the primary pathogen responsible for the most virulent form of bovine mastitis, posing the greatest challenge to dairy production in most countries [12,13,14]. Contaminated milk, meat, and other animal-derived foods can serve as vehicles for *S. aureus* [15,16,17,18], and when these foods are ingested by humans, *S. aureus* can secrete various enzymes and toxins (hemolysins, enterotoxins), leading to the onset of diseases (pneumonia, enteritis), facilitating the transmission of bacterial zoonoses, and posing a serious threat to public safety.

Currently, antibiotics remain the primary means of treating bacterial diseases caused by *S. aureus* infection. Antibiotics are commonly used in dairy cows, sheep, pigs, and seafood to prevent the spread of diseases caused by *S. aureus*. But they cause side effects, such as disrupting the balance of the internal bacterial flora, causing resistance issues, and damaging organs. Due to the abuse of antibiotics in humans, animal husbandry, and aquaculture, *S. aureus* has evolved multidrug-resistant strains, showing varying degrees of resistance to β -lactam, aminoglycoside, tetracycline, and macrolide antibiotics [19]. Among them, methicillin-resistant *Staphylococcus aureus* (MRSA) has become a significant public health challenge, and the World Health Organization has classified it as a high priority [20]. Meanwhile, livestock-associated methicillin-resistant *Staphylococcus aureus* (LA-MRSA), due to its highly infectious nature, extreme difficulty in treatment, and particular difficulty in prevention, is commonly transmitted between humans and animals [21]. Consequently, concerns over bacterial resistance have spurred the exploration of antibiotic alternatives, with bacteriotherapy for treating bacterial infections gaining increasing attention.

Probiotics and their metabolites are an important part of bacteriotherapy and are favored by many scholars because of their significant antibacterial activity and safety. Probiotics and their secreted bioactive metabolites exert a variety of beneficial effects on health and possess the ability to inhibit pathogens, making them effective candidates for novel antimicrobial drugs. Among these, lactic acid bacteria (LAB) are the most extensively studied and widely applied, serving as food preservatives or antimicrobial agents in various ways [22,23]. LAB consist of a diverse group of Gram-positive, coccoid, and rod-shaped bacteria, including species of *Lactobacillus*, *Leuconostoc*, *Streptococcus*, *Lactococcus*, and *Pediococcus* [24,25]. They are commonly used in the production of functional products and are considered safe and natural microorganisms [26]. Studies have shown that the cell-free supernatant of LAB exhibits inhibitory activity against pathogenic microorganisms such as *Listeria monocytogenes*, *Escherichia coli*, *Klebsiella pneumoniae*, and *Pseudomonas aeruginosa* [27,28,29]. *Streptococcus thermophilus* (*S. thermophilus*), *Lactiplantibacillus plantarum* (*L. plantarum*), *Bifidobacterium longum* subspecies *infantis* (*B. infantis*), *and Lacticaseibacillus rhamnosus* (*L. rhamnosus*) were the most common lactic acid bacteria, and their CFSs have antibacterial effects on a variety of pathogenic bacteria [30,31,32,33]. However, it remains unclear whether the CFSs of *S. thermophilus* IFFI 6038, *L. plantarum* ATCC 8014, *B. infantis* CICC 6099, and *L. rhamnosus* ATCC 7469 have inhibitory effects on *S. aureus*.

Therefore, the purpose of this study is to demonstrate the potential applications of CFSs derived from spent culture media of *L. plantarum*, *L. rhamnosus*, *S. thermophilus,* and *B. infantis*. The focus of this study is to evaluate the antibacterial activity of CFSs from *L. plantarum*, *L. rhamnosus*, *S. thermophilus*, and *B. infantis* against *S. aureus*, as well as their mechanisms of antibacterial action. This study will provide evidence for the potential application of CFSs of *S. thermophilus* IFFI 6038, *L. plantarum* ATCC 8014, *B. infantis* CICC 6099, and *L. rhamnosus* ATCC 7469 in preventing *S. aureus* contamination.

## 2. Materials and Methods

### 2.1. Bacterial Strains and Culture Conditions

The probiotic strains *Streptococcus thermophilus* IFFI 6038, *Lactiplantibacillus plantarum* ATCC 8014, *Lacticaseibacillus rhamnosus* ATCC 7469, and *Bifidobacterium longum* subspecies *infantis* CICC 6099 were preserved by the China Key Laboratory of Bovine Disease Control in Northeast China, Heilongjiang Bayi Agricultural University. The strains were inoculated into MRS medium (De Man, Rogosa and Sharpe medium, CM187, Beijing Land Bridge technology Co., Ltd., Beijing, China) broth and activated in incubators at 37 °C. *Staphylococcus aureus* BNCC 186335 preserved in our laboratory was cultured in CAMHB broth (Cation-adjusted Mueller-Hinton broth, HB6231-1, Qingdao hopebio technology Co., Ltd., Qingdao, China) using an incubator at 37 °C. All bacterial strains were stored at −80 °C before culturing.

### 2.2. Preparation of CFSs from L. plantarum, L. rhamnosus, S. thermophilus, and B. infantis

The cell-free supernatants were prepared according to methods described in previous reports with minor modifications [34]. The activated strains (10^8^ CFU/mL) were diluted in fresh MRS at a ratio of 1:200 and incubated at 37 °C for 24 h [35]. Centrifuge the probiotic culture at 8000× *g* for 15 min at 4 °C. The supernatants collected were filtered and sterilized using a 0.22 μm filter (SYRINGE FILTER, Lanjie Ke Technology Co., Ltd., Beijing, BS-PES-22) and stored at −80 °C.

### 2.3. Determination of Antimicrobial Activity

The antibacterial ability of CFSs against *S. aureus* was determined in vitro by the agar pore diffusion assay based on the diameter of the inhibition zone [36]. Dilute the exponentially growing *S. aureus* to a bacterial suspension of approximately 6–7 × 10^7^ CFU/mL, and then draw 100 μL of the bacterial solution and evenly distribute it on the CAMHB plate with agar added. Then, Oxford cups with an inner diameter of 6 mm and a height of 10 mm were placed and filled with 200 μL of sterile CFS. An equal amount of sterilized MRS medium was used as the negative control. The plates were incubated in a constant temperature incubator at 37 °C for 12 h and 24 h, and then the diameter of the inhibition zone (in millimeters) was measured [37]. A broth microdilution method was used with some modifications. Briefly, *S. aureus* was diluted to 10^6^ CFU/mL. Then, 50 μL of bacterial suspension was mixed with four CFS aliquots (50 μL) in sterile 96-well plates. A total of 50 μL of bacterial suspension was mixed with CAMHB broth (50 μL) and MRS broth (50 μL) as control. The samples were incubated at 37 °C for 12 h and 24 h, and then their absorbance values at a wavelength of 620 nm were measured using Varioskan Lux (Thermo Fisher Scientific Co., Ltd., Waltham, MA, USA) [38].

### 2.4. CFS Stability Test

For the determination of storage stability, CFSs were stored at 25 °C, 4 °C, −20 °C, and −80 °C for 7 d, 14 d, and 21 d. The antibacterial activity of CFSs was determined by agar pore diffusion. For the thermal stability assay, CFSs were treated at 42 °C and 60 °C for 1 h, to study the antibacterial effect of CFSs.

### 2.5. Minimal Inhibitory Concentration (MIC) Assay

The MIC of the four CFSs refer to the previous research method [39]. The *S. aureus* in CAMHB liquid medium was diluted to 6–7 × 10^5^ CFU/mL, and then the diluted bacterial suspension was treated with MRS and CFSs (5%, 10%, 20%, 30%, and 40%) and incubated at 37 °C for 24 h. The absorbance at 620 nm was measured using Varioskan Lux (Thermo Fisher Scientific Co., Ltd., Waltham, MA, USA).

### 2.6. Time–Kill Assays

Previous research methods were referenced with some modifications [40]. The standard inoculum of *S. aureus* (6–7 × 10^4^ CFU/mL) was exposed to CFSs of *L. plantarum*, *L. rhamnosus*, *B. infantis*, and *S. thermophilus*. CAMHB and MRS were used for control samples. During static incubation at 37 °C, 100 μL was taken from the samples at predetermined intervals (0.5, 1, 2, 3, 4, 6, 8, 10, 12, and 24 h), serially diluted in phosphate-buffered saline (PBS; P1010, Solarbio, Beijing, China), and evenly distributed on the plates for incubation for 24 h. The viability of *S. aureus* was evaluated by CFU counting.

### 2.7. NaOH, Catalase and Proteinase K Treatment of Supernatant

According to the method in Section 2.3, the antibacterial test was carried out with *S. aureus* as the indicator bacteria and the untreated CFSs as the control group [41]. The initial pH values of the four CFSs ranged from 3.99 to 4.22. The treatment group CFS was the supernatant adjusted to pH = 7.0 with 1 mol/L NaOH. The four cell-free supernatants were added with 5 mg/mL catalase to make the final concentration of 1 mg/mL, and incubated at 37 °C for 1 h. The four CFSs were added with 10 mg/mL proteinase K to make the final concentration of 1 mg/mL, incubated at 37 °C for 3 h.

### 2.8. Determination of Extracellular Nucleic Acid and Protein Content

Overnight bacterial cultures were diluted to (6–7 × 10^5^ CFU/mL) and incubated in culture supplemented with CFS (5% and 10%, *v*/*v*) and incubated for 4 h and 6 h. Samples were centrifuged (4 °C, 5000× *g*, 10 min), and the values of the OD_260 nm_ and OD_280 nm_ supernatants were read using Varioskan Lux (Thermo Scientific, USA), respectively. Untreated bacterial cultures served as negative controls.

### 2.9. ATP Bioluminescence Assay

ATP levels in cultures of the selected pathogenic bacteria treated with various concentrations of CFSs were measured using a BacTiter-Lumi™ ATP Assay Kit (C0052S, Beyotime, Beijing, China) according to the manufacturer’s instructions. Bioluminescence measurements were obtained in triplicate for each sample. *S. aureus* cultured in MRS was used as a negative control. Luminescence was measured using Varioskan Lux (Thermo Fisher Scientific Co., Ltd., Waltham, MA, USA).

### 2.10. Live-Dead Cell Observation by Fluorescence Microscope

The LIVE/DEAD Bacterial Staining Kit with DMAO (N,N-dimethylaniline N-oxide) and PI (Propidium iodide) (C2030S, Beyotime, Beijing, China) was used to distinguish live and dead bacterial cells. DMAO makes both live and dead bacteria show green fluorescence. PI can only penetrate the membrane of damaged bacteria, causing dead bacteria to show red fluorescence. The bacteria treated with CFSs or MRS medium (control) for 4 h were stained in the dark for 30 min according to the manufacturer’s instructions. The cells were mounted on slides and evaluated by OLYMPUS IX73 (EVDENT, Tokyo, Japan).

### 2.11. Scanning Electron Microscopy (SEM) Analysis

*S. aureus* BNCC 186335 was treated with CFSs of *L. plantarum*, *L. rhamnosus*, *B. infantis*, and *S. thermophilus* for 6 h at 37 °C. Samples were centrifuged and washed three times with PBS. The bacteria were fixed at room temperature using electron microscope fixative (B0008, POWERFUL BIOLOGY, Wuhan, China) for 2 h, and then stored at 4 °C. After that, the bacteria were continuously dehydrated with increasing concentrations of ethanol (50%, 70%, 90% and 100%) and dried. The observation and photographing were carried out under SEM Zeiss Sigma 300 (Zeiss, Oberkochen, Germany).

### 2.12. Measurement of Antibiofilm Activity

Some modifications were made based on the previous research method for the determination of antibiofilm activity [42]. The overnight cultures of *S. aureus* (6–7 × 10^8^ CFU/mL) were seeded in 12-well plates, supplemented with 5% CFS during biofilm formation, and incubated for 48 h. The liquid in the well was discarded and they were washed three times with PBS. Biofilms were stained with 0.1% crystal violet, washed three times, and resuspended in 33% glacial acetic acid. Biofilms were quantified at OD_570 nm_ using Varioskan Lux (Thermo Fisher Scientific Co., Ltd., Waltham, MA, USA). The results obtained were standardized using the *S. aureus* group as the standard.

### 2.13. Statistical Analysis

All experiments were performed in triplicate, and the data were statistically analyzed by GraphPad Prism 10.1.2 software. Data were analyzed by one-way ANOVA and two-way ANOVA. Each value of the obtained results is the mean of three replicates ± standard deviation. The results were statistically significant at *p* < 0.01 and *p* < 0.001, denoted by ∗∗ and ∗∗∗, respectively.

## 3. Results

### 3.1. Inhibition of CFSs of L. plantarum, L. rhamnosus, B. infantis, and S. thermophilus on S. aureus

**Figure 1:**
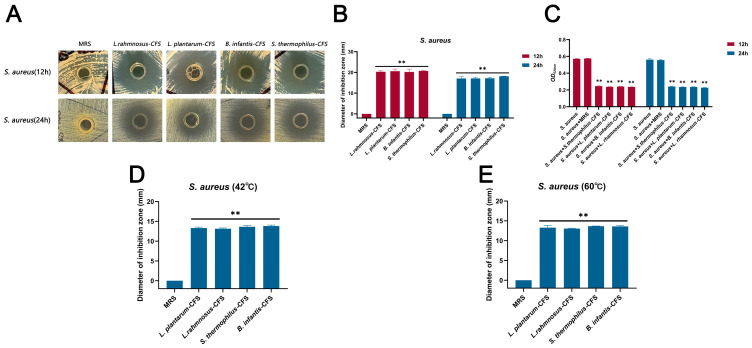
The antibacterial effect of CFSs of *S. thermophilus*, *B. infantis*, *L. plantarum*, and *L. rhamnosus* on *S. aureus*. (**A**) Oxford cup method for detecting the effect of CFSs of *S. thermophilus*, *B. infantis*, *L. plantarum*, and *L. rhamnosus* on *S. aureus.* (**B**) The diameter of the inhibition zone of the CFSs of *S. thermophilus*, *B. infantis*, *L. plantarum*, and *L. rhamnosus* against *S. aureus*. (**C**) Antibacterial activity of CFSs of *S. thermophilus*, *B. infantis*, *L. plantarum*, and *L. rhamnosus* against *S. aureus*. (**D**) The CFSs were placed at 42 °C for 1 h. (**E**) The CFSs were placed at 60 °C for 1 h. Each value represents the average of three independent measurements. Bars represent the standard deviation (*n* = 3). Two-way ANOVA test followed by Tukey–Kramer multiple comparisons test (** *p* < 0.01).

The CFSs of *L. plantarum*, *L. rhamnosus*, *B. infantis*, and *S. thermophilus* cultured for 24 h showed antibacterial activity against *S. aureus*, and the diameter of the inhibition zone exceeded 20 mm in 12 h and 17 mm in 24 h, respectively (Figure 1A–C). The CFSs of *L. plantarum*, *L. rhamnosus*, *B. infantis*, and *S. thermophilus* were not sensitive to heat treatment and retained significant antibacterial activity at 42 °C and 60 °C (** *p* < 0.01), and the diameter of the inhibition zone exceeded 13 mm (Figure 1D,E).

### 3.2. The Stability of CFSs of L. plantarum, L. rhamnosus, B. infantis, and S. thermophilus

**Figure 2:**
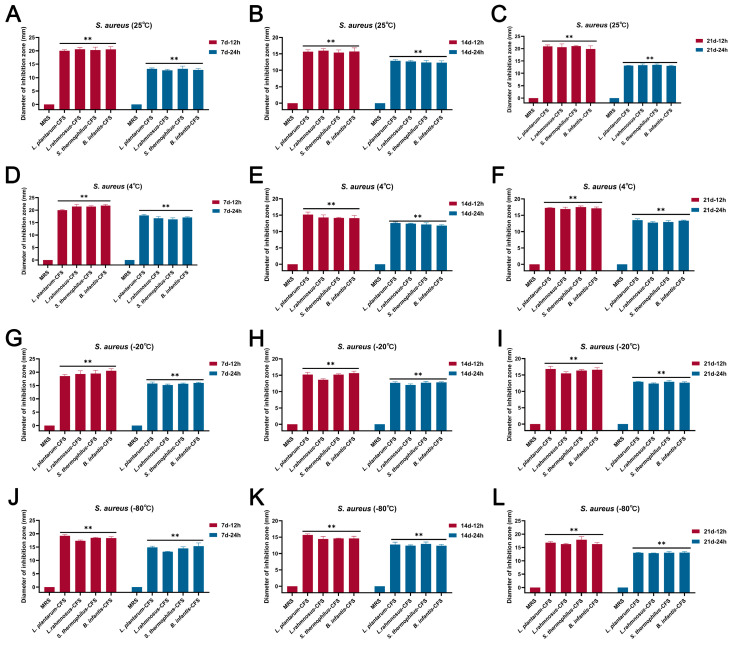
The effect of different storage conditions at different temperatures and times on the antibacterial activity of CFSs of *S. thermophilus*, *B. infantis*, *L. plantarum*, and *L. rhamnosus*. (**A**) 25 °C and 7 d. (**B**) 25 °C and 14 d. (**C**) 25 °C and 21 d. (**D**) 4 °C and 7 d. (**E**) 4 °C and 14 d. (**F**) 4 °C and 21 d. (**G**) −20 °C and 7 d. (**H**) −20 °C and 14 d. (**I**) −20 °C and 21 d. (**J**) −80 °C and 7 d. (**K**) −80 °C and 14 d. (**L**) −80 °C and 21 d. Each value represents the average of three independent measurements. Bars represent the standard deviation (*n* = 3). Two-way ANOVA test followed by Tukey–Kramer multiple comparisons test (** *p* < 0.01).

The results showed that the CFSs of *L. plantarum*, *L. rhamnosus*, *B. infantis*, and *S. thermophilus* stored at 25 °C, 4 °C, 20 °C, and −80 °C for 7 d, 14 d, and 21 d, respectively, still maintained bacteriostatic activity (Figure 2A–L).

### 3.3. Determination of MIC and Analysis of Killing Kinetics

**Figure 3:**
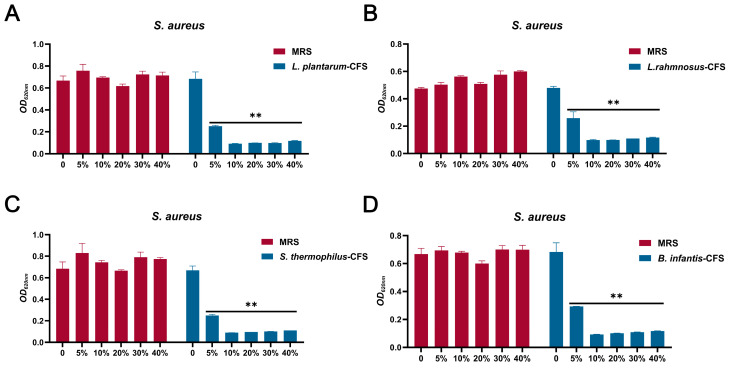
The MIC of CFSs of *S. thermophilus*, *B. infantis*, *L. plantarum*, and *L. rhamnosus*. (**A**) *L. plantarum.* (**B**) *L. rhamnosus*. (**C**) *S. thermophilus.* (**D**) *B. infantis.* Each value represents the average of three independent measurements. Bars represent the standard deviation (*n* = 3). Two-way ANOVA test followed by Tukey–Kramer multiple comparisons test (** *p* < 0.01).

**Figure 4:**
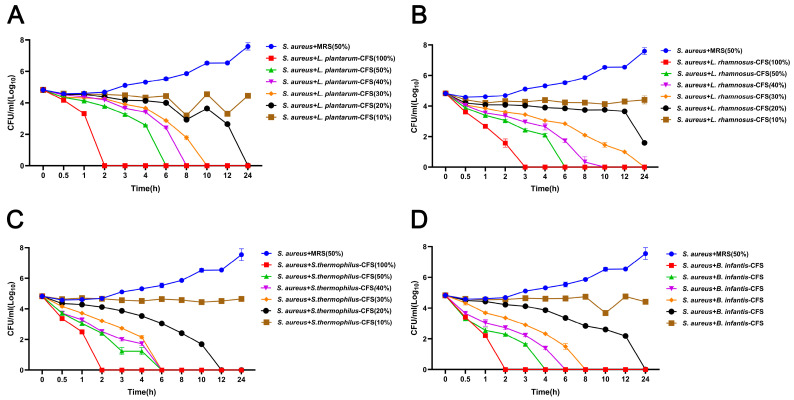
The effect of CFSs of *S. thermophilus*, *B. infantis*, *L. plantarum*, and *L. rhamnosus* on the time-killing curve of *S. aureus*. (**A**) *L. plantarum.* (**B**) *L. rhamnosus*. (**C**) *S. thermophilus.* (**D**) *B. infantis.* Each value represents the average of three independent measurements. Bars represent the standard deviation (*n* = 3).

The results showed that four CFS (10%, 20%, 30%, and 40%) treatments could significantly inhibit the growth of *S. aureus*. In contrast, the inhibitory effect of 5% CFS on *S. aureus* was lower than that of 10%, 20%, 30%, and 40% CFS (Figure 3A–D). The CFS of 100% *L. plantarum* could eradicate bacteria in 2 h, and the CFS of 50%, 40%, 30%, and 20% *L. plantarum* could eradicate *S. aureus* in 6 h, 8 h, 10 h, and 24 h, respectively (Figure 4A). The CFS of 100% *L. rhamnosus* killed *S. aureus* within 3 h, while the CFS of 30% *L. rhamnosus* could kill *S. aureus* within 24 h (Figure 4B). The CFS of 100% *B. infantis* and *S. thermophilus* could eradicate *S. aureus* in 2 h, while the CFS of 50%, 40%, and 30% *S. thermophilus* could kill *S. aureus* in 6 h, and the CFS of 20% *B. infantis* could eradicate bacteria in 24 h (Figure 4C,D).

### 3.4. Analysis of Antibacterial Substances in CFSs

**Figure 5:**
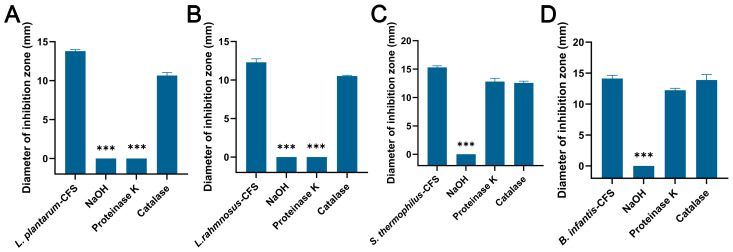
The effects of proteinase K, NaOH, and catalase on the antibacterial activity of CFSs of *S. thermophilus*, *B. infantis*, *L. plantarum*, and *L. rhamnosus*. (**A**) *L. plantarum.* (**B**) *L. rhamnosus*. (**C**) *S. thermophilus.* (**D**) *B. infantis.* Each value represents the average of three independent measurements. Bars represent the standard deviation (*n* = 3). One-way ANOVA test followed by Tukey–Kramer multiple comparisons test (*** *p* < 0.001).

The CFSs of *L. plantarum* and *L. rhamnosus* treated with NaOH and proteinase K failed to produce an inhibition zone, while the control group and catalase group formed an obvious inhibition zone (Figure 5A,B). For the CFSs of *B. infantis* and *S. thermophilus*, only the NaOH group failed to produce an inhibition zone, while other groups showed obvious inhibition zones (Figure 5C,D).

### 3.5. The Effect of CFSs on the Cell Wall and Membrane of S. aureus

**Figure 6:**
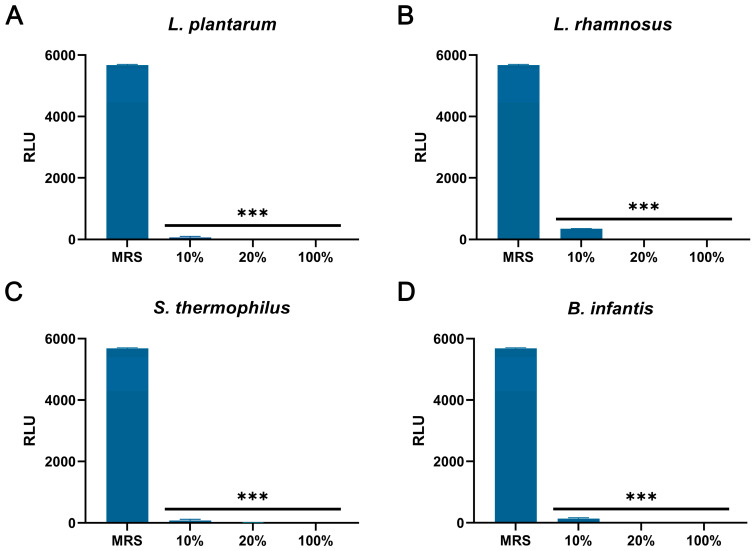
The effect of CFSs of *S. thermophilus*, *B. infantis*, *L. plantarum*, and *L. rhamnosus* on intracellular ATP levels in *S. aureus*. (**A**) *L. plantarum.* (**B**) *L. rhamnosus*. (**C**) *S. thermophilus.* (**D**) *B. infantis.* Each value represents the average of three independent measurements. Bars represent the standard deviation (*n* = 3). One-way ANOVA test followed by Tukey–Kramer multiple comparisons test (*** *p* < 0.001).

**Figure 7:**
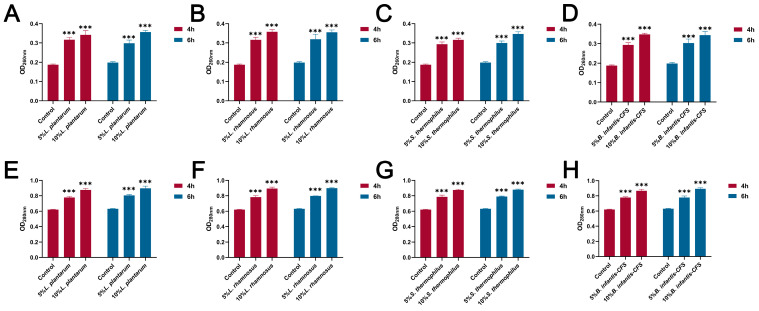
The effect of CFSs of *S. thermophilus*, *B. infantis*, *L. plantarum*, and *L. rhamnosus* on extracellular nucleic acid and protein content in *S. aureus* (4 h and 6 h). (**A**) The effect of CFS of *L. plantarum* on extracellular nucleic acid content in *S. aureus*. (**B**) The effect of CFS of *L. rhamnosus* on extracellular nucleic acid content in *S. aureus*. (**C**) The effect of CFS of *S. thermophilus* on extracellular nucleic acid content in *S. aureus*. (**D**) The effect of CFS of *B. infantis* on extracellular nucleic acid content in *S. aureus*. (**E**) The effect of CFS of *L. plantarum* on extracellular protein content in *S. aureus*. (**F**) The effect of CFS of *L. rhamnosus* on extracellular protein content in *S. aureus*. (**G**) The effect of CFS of *S. thermophilus* on extracellular protein content in *S. aureus*. (**H**) The effect of CFS of *B. infantis* on extracellular protein content in *S. aureus*. Each value represents the average of three independent measurements. Bars represent the standard deviation (*n* = 3). Two-way ANOVA test followed by Tukey–Kramer multiple comparisons test (*** *p* < 0.001).

As shown in Figure 6A–D, compared with the control group, the CFSs of *L. plantarum*, *L. rhamnosus*, *B. infantis*, and *S. thermophilus* significantly reduced the intracellular ATP of *S. aureus* (*p* < 0.001). In addition, the content of extracellular nucleic acids and proteins in *S. aureus* treated with CFSs of *L. plantarum*, *L. rhamnosus*, *B. infantis*, and *S. thermophilus* for 4 h and 6 h was significantly increased compared with the control group (*p* < 0.001) (Figure 7A–H).

### 3.6. The Effect of CFSs of L. plantarum, L. rhamnosus, B. infantis, and S. thermophilus on the Integrity of S. aureus Cell Membrane

**Figure 8:**
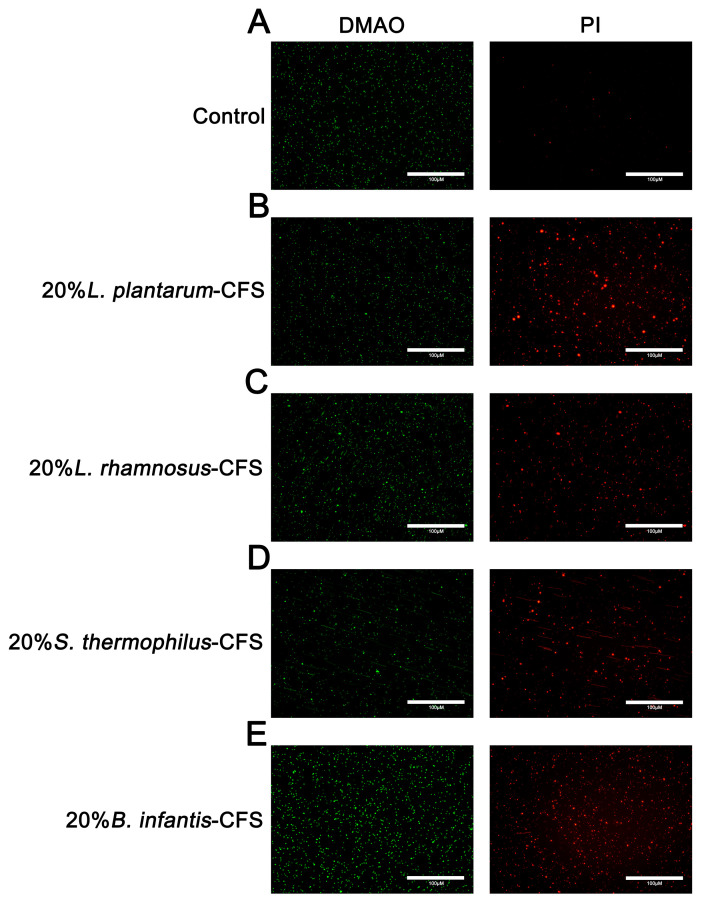
The representative fluorescence images of LIVE/DEAD Bacterial Staining of *S. aureus* in CFSs of *S. thermophilus*, *B. infantis*, *L. plantarum*, and *L. rhamnosus*. (**A**) Control. (**B**) 20% *L. plantarum.* (**C**) 20% *L. rhamnosus*. (**D**) 20% *S. thermophilus* (**E**) 20% *B. infantis.* Red fluorescence represents dead or membrane-damaged bacteria, while green represents live and dead bacteria.

The results showed that untreated *S. aureus* emitted green fluorescence but almost no red fluorescence. After treating *S. aureus* with CFSs of *L. plantarum*, *L. rhamnosus*, *B. infantis*, and *S. thermophilus*, the bacteria showed red fluorescence (Figure 8A–E).

### 3.7. The Effect of CFSs of L. plantarum, L. rhamnosus, B. infantis, and S. thermophilus on the Morphology of S. aureus

**Figure 9:**
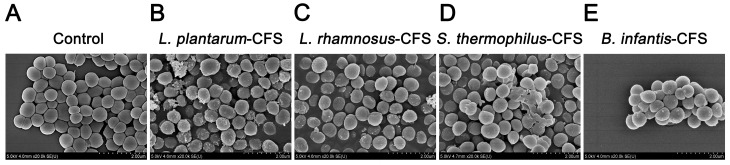
Live–dead cells observed by field emission scanning electron micrograph of *S. aureus* treated with CFSs of *S. thermophilus*, *B. infantis*, *L. plantarum*, and *L. rhamnosus*. (**A**) Control. (**B**) *L. plantarum.* (**C**) *L. rhamnosus*. (**D**) *S. thermophilus.* (**E**) *B. infantis*.

The *S. aureus* in the control group showed a round and smooth spherical shape at a magnification of 20,000× (Figure 9A). When the CFSs of *L. plantarum*, *L. rhamnosus*, *B. infantis*, and *S. thermophilus* was used to treat *S. aureus*, the membrane surface of *S. aureus* was damaged, changing from smooth to rough and accompanied by unevenness (Figure 9B–E).

### 3.8. The Inhibitory Effect of CFSs of L. plantarum, L. rhamnosus, B. infantis, and S. thermophilus on the Biofilm Formation of S. aureus

**Figure 10:**
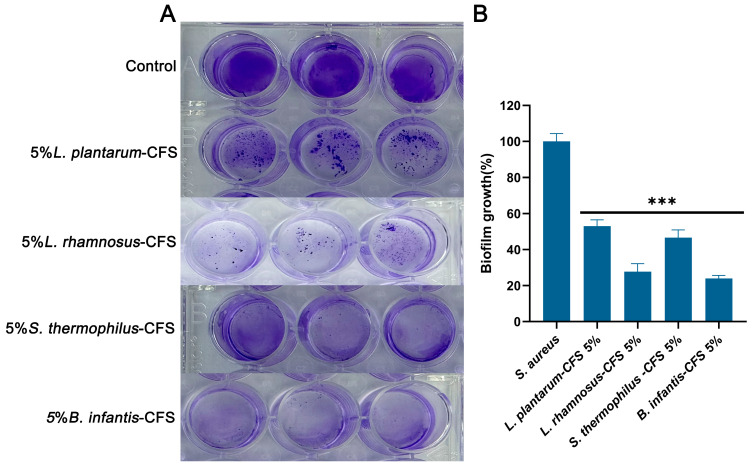
The effect of CFSs of *S. thermophilus*, *B. infantis*, *L. plantarum*, and *L. rhamnosus* (5%) on the biofilm of *S. aureus*. (**A**) Crystal violet staining results. (**B**) Quantitative analysis of biofilm biomass. Each value represents the average of three independent measurements. Bars represent the standard deviation (*n* = 3). One-way ANOVA test followed by Tukey–Kramer multiple comparisons test (*** *p* < 0.001).

As shown in Figure 10A, the control group formed a significant biofilm under crystal violet staining. After treatment with 5% CFS of *L. plantarum*, *L. rhamnosus*, *B. infantis*, and *S. thermophilus*, the biogenesis of *S. aureus* was significantly reduced compared to the control group (*p* < 0.001) (Figure 10B). After quantifying the biofilm, compared with the control group, the CFS treatment of *L. plantarum*, *L. rhamnosus*, *B. infantis*, and *S. thermophilus* reduced the formation of *S. aureus* biofilm by 47.07%, 72.32%, 76.11%, and 53.38%, respectively. These results indicated that four types of CFS (5%, *v*/*v*) could influence the biofilm stage of *S. aureus*.

## 4. Discussion

Humans may suffer damage to their health and even die by coming into contact with and consuming animals or foods infected with *S. aureus*. As is well known, *S. aureus*, as a foodborne pathogenic microorganism, is widely distributed in milk, meat, and aquatic products [43]. Antibiotics are effective in killing *S. aureus* and remain the primary method of antibacterial treatment [44]. However, the long-term use of antibiotics can easily induce bacteria to evolve drug-resistant genes, making the prohibition of antibiotic abuse imperative. Simultaneously, this is also a major reason why numerous researchers are exploring whether the cell-free supernatant of probiotics possesses antibacterial activity. LAB has long been widely recognized and applied in dairy products such as milk, milk powder, and cheese through labels that are safe and beneficial to human health [45]. However, their ability as natural antimicrobial substances is a current research hotspot. Existing evidence indicates that LAB inhibits the survival and replication of pathogenic microorganisms by secreting metabolites with antimicrobial effects [46]. In our study, we revealed and explored the effects and antibacterial mechanisms of CFS produced by *L. plantarum*, *L. rhamnosus*, *S. thermophilus*, and *B. infantis* on *S. aureus*. Previous studies have shown that the CFS of *Bifidobacterium longum* FB1-1 exhibited significant bactericidal effects against carbapenem-resistant *Klebsiella pneumoniae* (CRKP) and restricted the dissemination of resistance genes and the expression of virulence genes (*bla_KPC*, *uge*, and *fim_H*) in CRKP, thereby inhibiting the transfer of CRKP resistance [30]. One interesting finding is that the supernatant without *L. plantarum* O24 exhibited effective antibacterial activity against *Listeria monocytogenes* and *Salmonella Typhimurium*, and its good antioxidant properties can serve as an alternative to food preservatives [31]. The current study found that the CFS of *L. rhamnosus* SCB0119 affected the transcription of genes related to fatty acid degradation and amino acid biosynthesis in *Escherichia coli*, leading to alterations in the morphological structure of *E. coli* [32]. Another important finding was that the CFS of *S. thermophilus* M18 altered the polysaccharide and lipid content and composition of *Pseudomonas aeruginosa*, which reduced the growth of *Klebsiella pneumoniae* and enhanced the antibiotic sensitivity of these two pathogenic bacteria [33]. However, to date, the antibacterial mechanisms of CFSs of *L. plantarum*, *L. rhamnosus*, *S. thermophilus*, and *B. infantis* remain unclear.

Interestingly, our study demonstrated that all four tested CFSs maintained a sustained and rapid bactericidal activity against *S. aureus*, with a significant reduction in bacterial load observed starting from 30 min. This finding is consistent with that of other researchers, who have observed that pathogenic microorganisms were strongly and rapidly killed by the CFSs of probiotics [40]. Of course, the smaller initial inoculum may have affected the determination of the bactericidal activity of CFS. Meanwhile, we observed that compared with the experimental group treated with CFSs, the number of bacteria in the control group treated with MRS was significantly increased from 3 h, and the total number of bacteria was greater than 10^7^ CFU/mL at 24 h. In addition, the CFS of *Lactobacillus sakei* NRRL B-1917 reduced the quantities of *Escherichia coli* and *Listeria monocytogenes* [47]. In addition, there is evidence that the CFSs of *Lactobacillus gasseri* 1A-TV, *L. fermentum* 18A-TV, and *L. crispatus* 35A-TV have strong bactericidal effects on *Streptococcus agalactiae*, *Escherichia coli*, *Klebsiella pneumoniae*, and *S. aureus* [48]. The time used by CFSs produced by *L. plantarum*, *L. rhamnosus*, *S. thermophilus*, and *B. infantis* in this study to kill bacteria is lower than that used by CFSs in the above study. Therefore, CFSs produced by *L. plantarum*, *L. rhamnosus*, *S. thermophilus*, and *B. infantis* in this study have certain advantages in killing *S. aureus*. This might be related to the metabolites contained in the CFSs of probiotics. These results suggest that the CFSs of different probiotics may share a common way of killing pathogenic bacteria. They can damage the integrity of the cell wall and membranes of pathogenic microorganisms, increasing the release of malondialdehyde, alkaline phosphatase, and intracellular substances [49,50,51,52].

Studies have shown that the CFSs of *Lactobacillus sakei* NRRL B-1917 retained their antibacterial activity after storage at 15 °C, 25 °C, and 35 °C for a period of time [53]. In addition, the CFS produced by *Lactobacillus coryniformis* 7841 still maintained a certain antibacterial effect after being treated at 37 °C, 50 °C, 70 °C, 90 °C, 100 °C, and 121 °C for 20 min, respectively [41]. The CFSs of *L. plantarum*, *L. rhamnosus*, *S. thermophilus*, and *B. infantis* were found to be stable and well tolerated. Compared with other studies, this study explored the antibacterial activity of CFSs from *L. plantarum*, *L. rhamnosus*, *S. thermophilus*, and *B. infantis* at 4 °C, −20 °C, and −80 °C and expanded the reference potential of four CFSs in transportation and storage conditions. This was not included in the previous research institute. Therefore, the four types of CFS have the potential to be added to foods that require long-distance transportation, low-temperature refrigeration, and freezing, which helps reduce bacterial growth in food. This undoubtedly reduces food spoilage caused by bacterial contamination, decreases economic losses in the livestock and food industries, and ensures food safety.

An unavoidable problem is that, regardless of the storage temperature of the CFS, the diameter of the inhibition zone of a CFS against *S. aureus* will be smaller over time than it was when it was just prepared. This is most likely related to the loss of antimicrobial components in CFSs. CFSs from *L. plantarum* and *L. rhamnosus* may exert antimicrobial effects through organic acids and protein components. CFSs produced by *S. thermophilus* and *B. infantis* may inhibit *S. aureus* activity through organic acids. Of course, NaOH and proteinase K experiments can only preliminarily verify the involvement of organic acids and protein components in CFSs in the antibacterial activity against *S. aureus*. The main antibacterial substances in the four CFSs still need to be analyzed by nano liquid chromatography coupled with tandem mass spectrometry analysis and other techniques. Studies have shown that the antibacterial activity of cell-free supernatants produced by various lactic acid bacteria mainly depends on the content of organic acids [54]. This report supported our findings. Meanwhile, *L. plantarum* FB-2, *L. rhamnosus* L60, and *Lactobacillus fermentum* L23 exerted inhibitory effects on pathogenic bacteria through bacteriocins extracted from CFSs [51,55]. Furthermore, research has confirmed that the organic acids and bacteriocins contained in CFSs could alter bacterial morphology, causing damage to the cell membrane structure of pathogenic bacteria and leakage of intracellular substances [56,57]. And it is worth noting that the CFSs produced by the four probiotics could damage the integrity of the cell membrane of *S. aureus* and cause damage to the cell morphology, lead to the release of nucleic acids and proteins, and reduced the intracellular ATP levels. This is similar to the antimicrobial mechanisms reported by other studies for organic acids and bacteriocins in CFSs. These results supported the involvement of organic acids and protein components in the four CFSs in the antibacterial action against bacteria.

After *S. aureus* attaches to biological tissues or abiotic surfaces, it forms a biofilm through polysaccharide intercellular adhesins, staphylococcal a protein, fibronectin binding protein, and biofilm-related proteins and enhances drug resistance and evades host immunity [58,59,60,61]. This ability to live in clusters reduces the sensitivity of *S. aureus* to antimicrobial substances, and these drugs experience difficulty in penetrating multiple layers of bacteria to destroy the deepest bacteria. Therefore, it is very important to screen out antibacterial agents that can destroy biofilm. At present, most evidence shows that cell-free supernatants have anti-biofilm properties, and the transcription of biofilm-related genes *agrA*, *prfA*, *flaA*, and *plcB* of pathogenic bacteria is inhibited by CFSs [62,63]. In our study, we found that all four 5% CFSs could inhibit the biofilm formation of S. aureus during the biofilm formation stage.

We note that there are certain shortcomings to this study. We found that four CFSs affect the growth of *S. aureus* biofilms in vitro, but we have not determined the changes in the gene expression of *S. aureus* biofilm or stress genes. Moreover, we have not conducted the antibacterial activity tests of CFSs on various strains of *S. aureus*. Meanwhile, the antimicrobial constituents (organic acids and protein components) in CFSs have not been directly characterized. Such experiments are planned in the next phase of our research. In further studies, we will use multi-omics analysis of the four CFSs to characterize their antimicrobial substances. In addition, to further clarify the antimicrobial activity of CFSs, we will conduct studies using CFSs in cell and in vitro models to evaluate its safety and feasibility. Such studies would also allow for more in-depth analyses of the interactions between the four CFSs and *S. aureus* and could have great significance in the development of antibiotic substitutes.

## 5. Conclusions

This study identified the antibacterial activity of CFSs produced by *S. thermophilus*, *B. infantis*, *L. plantarum*, and *L. rhamnosus*, and their CFSs showed significant inhibitory effects against *S. aureus*. At the same time, the antibacterial mechanism of CFSs of *S. thermophilus*, *B. infantis*, *L. plantarum*, and *L. rhamnosus* was further explored. The four types of CFS could inhibit the formation of bacterial biofilms, disrupt the integrity of cell membranes, lead to the release of cellular contents, cause significant changes in cell morphology, and reduce ATP levels, ultimately resulting in the death of *S. aureus*. In addition, the CFSs produced by *S. thermophilus*, *B. infantis*, *L. plantarum*, and *L. rhamnosus* showed good stability and tolerance and continued to exert antibacterial activity after being stored at different temperatures for a period of time. Overall, the above results indicate that the CFSs of *S. thermophilus*, *B. infantis*, *L. plantarum*, and *L. rhamnosus* have potential antibacterial and anti-biofilm potential and also provide new insights into the development of new formulas for the treatment of *S. aureus* infection with natural compounds.

## Data Availability

The original contributions presented in this study are included in the article. Further inquiries can be directed to the corresponding authors.

## Abbreviations

The following abbreviations are used in this manuscript:

*S. aureus*	*Staphylococcus aureus*
CFS	cell-free supernatant
*L. plantarum*	*Lactiplantibacillus plantarum*
*L. rhamnosus*	*Lacticaseibacillus rhamnosus*
*S. thermophilus*	*Streptococcus thermophilus*
*B. infantis*	*Bifidobacterium longum subspecies infantis*
